# Prevalence and long-term predictors of persistent chronic widespread pain in the general population in an 11-year prospective study: the HUNT study

**DOI:** 10.1186/1471-2474-15-213

**Published:** 2014-06-20

**Authors:** Ingunn Mundal, Rolf W Gråwe, Johan H Bjørngaard, Olav M Linaker, Egil A Fors

**Affiliations:** 1 Department of Neuroscience, Faculty of Medicine, Norwegian University of Science and Technology, 7491 Trondheim, Norway; 2 Outpatient Department of Psychiatry, Kristiansund Hospital, Møre and Romsdal Hospital Trust, Kristiansund, Norway; 3 Department of Research and Development, Addiction Medicine, St. Olav’s University Hospital, Trondheim, Norway; 4 Department of Public Health and General Practice, Faculty of Medicine, Norwegian University of Science and Technology, Trondheim, Norway; 5 Forensic Department and Research Centre Bröset, St Olav’s University Hospital, Trondheim, Norway; 6 Department of Psychiatry, Department of Research and Development, St Olavs University Hospital, Trondheim, Norway; 7 Department of Psychiatry, St Olavs University Hospital, Trondheim, Norway

**Keywords:** Chronic widespread pain, Anxiety, Depression, Alcohol, Prospective cohort study, General population

## Abstract

**Background:**

Chronic widespread pain (CWP) is common and associated with prominent negative consequences. The aim of this study was to assess the prevalence of persistent CWP in an 11-year prospective cohort study in the general population, and to examine anxiety, depression, alcohol use, poor sleep, body mass index (BMI) and chronic disease, along with demographic, lifestyle and other health-related variables as possible predictors for the assumed CWP persistence.

**Methods:**

CWP was defined as having pain at three or more predefined sites (involving the trunk and upper and lower limbs) for at least three months in the last year. We used a Norwegian general population cohort of 28,367 individuals who responded to both the second (1995–1997) and the third (2006–2008) waves of the Nord-Trøndelag Health Study (HUNT2 and HUNT3, respectively). Data were analysed with logistic regression models.

**Results:**

CWP prevalence in HUNT2 was 17%. Of those reporting CWP in HUNT2, 53% still reported CWP at follow-up in HUNT3. Adjusted analyses revealed that depression and alcohol consumption were not substantially associated with the 11-year prospective CWP outcome. Poor sleep, obesity and chronic disease predicted persistent CWP, and being male and/or 60 years or older was protective.

**Conclusions:**

This cohort study revealed that nearly half of the participants with baseline CWP resolved from CWP 11 years later. Among those whose CWP did not resolve, obesity, sleeping problems and chronic disease predicted CWP persistence, while aging and male sex was protective. Anxiety, mixed anxiety and depression, former smoking, and overweight were weakly associated, while depression, moderate exercise, and alcohol use were not associated with persistent CWP.

## Background

Chronic pain is common in the general population with a prevalence ranging from 20% to 55% [1-5]. Chronic pain persists beyond three months and is characterized by repeated or continuous pain episodes [6]. Other pain dimensions are quantitative (intensity) [7,8], qualitative (burning, pressing pain) [9], and spatial measures (whether the pain is local, regional or widespread) [10-12]. Chronic widespread pain (CWP) is an important measure of the global burden of pain [1,13], with a reported prevalence of 4% to 18% [14,15]. These various aspects of chronic pain have been assessed extensively and studied together as one entity, but rarely separately. Wolfe et al. [16] have defined CWP as pain in the left and right sides of the body, above and below the waist, plus pain in the axial skeleton. CWP seems to be more prevalent in females than in males [17,18], but it appears to be constant between age groups, and is associated with several complex medical conditions [19] and complaints, e.g. depression, anxiety, poor sleep, appetite disturbances and fatigue [20].

Cross-sectional studies have demonstrated that different pain characteristics are interrelated [21,22]. They have examined the relation between CWP prevalence, demographic variables, lifestyle factors, and self-reported health information [21,23,24], and have also reported that multisite pain problems are significantly associated with mood and anxiety disorders, but not with alcohol abuse [25]. These factors are also assessed as risk factors of CWP onset in population-based prospective studies [15,26-28]. A recent review of risk factors associated with transitioning from regional pain to CWP suggested that females, those of older age, and those with a family history of pain and depressed mood, were all at higher risk [29].

A few population-based prospective studies have examined predictive factors of CWP persistence associated with CWP prevalence, suggesting that some 35% of those with initial CWP, are likely to experience persistence when they are females, of older ages, or exhibit additional physical or psychological symptoms [30,31]. In a three-year follow-up study of pain distribution and risk factors, Bergman and colleagues [32] found that the overall prevalence of CWP was persistent over a three-year period, where 57% with initial CWP continued to have CWP at follow-up. While CWP persistence was predicted by the number of painful regions at baseline, as well by being an immigrant, having personal social support and drinking alcohol weekly seemed to be protective against persistence [32]. A 5.5 year prospective study of a Norwegian female population discovered that 61% with initial CWP continued to have CWP at follow-up [33]. Psychological symptoms were only assessed in one of these studies [30]. We are not aware of any large prospective population-based study investigating mood, chronic disease, lifestyle and demographic factors together as predictors of long-term CWP persistence. In view of the fact that CWP is closely associated with negative bio-psychosocial consequences [34] and mostly assessed in clinical samples, it is important to obtain knowledge of the overall impact of factors that predict CWP persistence in the general population.

The aim of this study was to 1) assess the prevalence, and 2) analyse possible predictive health-related, lifestyle and demographic factors as long-term predictive factors of CWP persistence. We hypothesized that (i) anxiety, depression, poor sleep, smoking and BMI ≥ 25 kg/m^2^ are predictors of CWP persistence and that (ii) young age, male sex, alcohol consumption and moderate exercise are protective factors.

## Methods

### Study population and baseline

This study is based on a large representative Norwegian cohort of individuals attending the second and third waves of the Nord-Trøndelag Health Study (HUNT2 and HUNT3, respectively). The HUNT study (“Helseundersøkelsen i Nord-Trøndelag”), designed as a longitudinal cohort study consisting of three health surveys, was carried out three times at 11 year intervals, in which all residents aged 20 years and older in Norway’s Nord-Trøndelag County were invited to participate. The first survey (HUNT1) was conducted between 1984 and 1986, while HUNT2 and HUNT3 were performed in 1995–1997 and 2006–2008, respectively. HUNT1 was designed primarily to cover four sub-studies regarding hypertension, diabetes, lung diseases and quality of life [35], while the scientific programme in HUNT2 was extended to encompass large public health issues, including mental health and chronic musculoskeletal pain [36]. Pain data were not collected from HUNT1 and thus could not be included in this study. The health data in all the studies were collected in the same way, by interviews, self-administrated questionnaires and clinical examinations. The majority of participants in all HUNT surveys were middle aged or elderly (50 – 79 years) and female [35].

**Figure 1:**
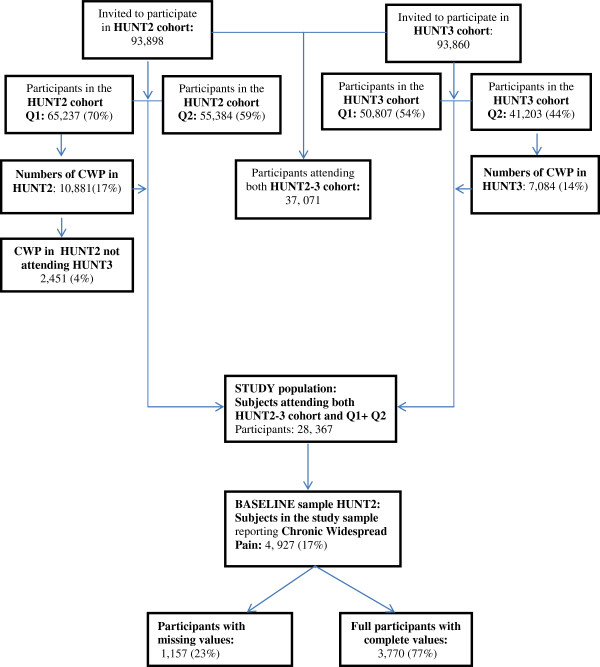
**Flowchart showing the inclusion of participants in the HUNT2 and HUNT3 study (Q1), the study population (participants responding to both Q1 and Q2 in HUNT2 and HUNT3) and baseline sample (chronic widespread pain in HUNT2) calculated from the study population.** Q1: Questionnaire 1, Q1: Questionnaire 2.

This study includes those 20 years and older in age (reaching 20 years during the year of screening in their municipality) who attended both HUNT2 and HUNT3 [35]. Of those invited in HUNT2, a total of 65,237 (70%) participated. In HUNT3 a total of 93,860 were eligible for participation, where 50,807 (54%) participated [36]. Participation in each survey was defined as having filled in questionnaire 1 (Q1), and a written consent form was delivered with Q1 when the participants attended the health examination sites. We included data collected from Q1, Q2 and the health examination (such as body mass index), and both questionnaires were distributed in HUNT2 and HUNT3. Whereas Q1 was a self-reporting questionnaire scored at home before attending the basic health examination, Q2 was handed out after the clinical examination and was to be completed at home and returned by mail [36].Because the baseline questions related to pain were asked in Q1 in HUNT2 and the follow-up questions were asked in Q2 in HUNT3, it was necessary to include all subjects responding to both questionnaires in both surveys. Hence, 28,367 individuals – 16,260 (57%) females and 12,107 (43%) males – responded to Q1 and Q2 in both HUNT2 and HUNT3, and these individuals constituted the study population. Of these, 4,927 participants reported CWP in HUNT2 and thus provided the baseline sample (see Figure 1).

### Dependent variable – CWP as reported in HUNT3

The chronicity of musculoskeletal pain was determined by the participants responding positively with a “yes” on the following question from the validated Standardized Nordic Questionnaire (SNQ): “During the last year, have you had pain and/or stiffness in your muscles and limbs that has lasted for at least 3 consecutive months?” [37]. Those reporting chronic pain were invited to specify their pain location(s) on a map of the human outer body [38]. The location included the following nine possible pain sites: neck, shoulder, elbow, hand/wrist, upper back, lower back, hip, knee and ankle/foot. If the participants responded “yes” to the introductory question but did not respond to the follow-up location question, they were still considered as having chronic pain, albeit not in the pictured area. The outcome variable CWP was constructed according to the Standardized Nordic Questionnaire and the American College of Rheumatology’s (ACR) defined criteria for CWP [39]. CWP in the present study was defined as having chronic pain in all the following three major body areas: the trunk (i.e. at least one of the following locations: neck, lower back, and/or upper back), lower limbs (i.e. at least one of the following: ankles, feet, knees and/or hips), and upper limbs (i.e. at least one of the following: shoulders, elbows and/or hands). As in the original version of SNQ, the subjects in HUNT3 could indicate bilateral bodily pain, but this information was omitted because of the lack of ability to indicate bilateral pain in HUNT2, where the baseline CWP variable was computed. According to ACR’s abbreviated definition, widespread pain is described as axial pain, left- and right-side pain, and upper- and lower-segment pain [39]. Pain in the left or right shoulder and buttock was considered as pain in each side, and lower-back pain was considered as lower-segment pain [16]. Thus, CWP was defined as the presence of pain in all four quadrants of the body. We analysed the CWP as a categorical variable, with categories 0 and 1.

### Possible predictors

The independent variables were sex and baseline information on age, separate anxiety and depression measures from the Hospital Anxiety and Depression Scale (HADS), alcohol use, insomnia and body mass index (BMI). Variables also included smoking status, marital status, education, exercise, and chronic disease, all of which were observed in the HUNT2 period. HADS is a well-established and validated 14-item, self-rating questionnaire that aims to detect general symptoms of anxiety and depression. It comprises seven items for depression (HADS-D) and seven for anxiety (HADS-A), in which each item includes a four-point Likert-scale response category (0–3) [40,41]. A mixed HADS variable was constructed and categorized into four groups: neither anxiety nor depression, pure depression, pure anxiety, and both depression and anxiety. The cut-off score of ≥ 8 was set to define the caseness of having anxiety or depression, and a cut-off score of ≥ 8 on each indicated the caseness of having both anxiety and depression [40,42]. In this study we wanted to examine the concurrence of anxiety and/or depression symptoms in relation to the scope of musculoskeletal pain rather than identifying the actual caseness of having anxiety and/or depression. However, the cut-offs were set to mark the borderline of depression or anxiety or both, even though a structured clinical interview, e.g. SCID-I, is the golden standard in clinical settings [43]. Because data from alcohol screening tools could not adequate reflect problems with alcohol, we decided to use data related to the frequency of alcohol use. The frequency of alcohol consumption was identified by two questions: “Concerning alcohol, are you a non-drinker?” and “how many times a month do you normally drink alcohol?” Responses were categorized as 1) abstainer, 2) drinking 0 times a month, 3) drinking 1–7 times a month, or 4) drinking 8 times or more a month. Drinking alcohol 0 times a month indicates a person that rarely drinks alcohol, but is not the same as a non-drinker by principle.

The influences that sleeping problems had on the CWP outcome were examined by using three questions from Q2, each related to non-restorative sleep, difficulties of sleep onset, and problems of maintaining sleep. Non-restorative sleep was identified by asking “how often do you suffer from insomnia?”, where the response options included “never or a few times a year”, “1–2 times a month”, “about once a week” and “more than once a week”. Problems of sleep onset and maintaining sleep were measured by two questions: “Have you had difficulty falling asleep in the last month?” and “during the last month, have you woken too early and not been able to get back to sleep?” Response options included “almost every night”, “often”, “now and again” and “never”, where the responses “almost every night” and “often” were defined as problems (1) and “now and again” and “never” as no problems (0). The three variables of “non-restorative sleep”, “sleep onset” and “maintaining sleep” were combined into one variable reflecting sleeping problems and categorized as “no problem” or “problem” (i.e. reporting 1 or more sleeping problems on the 3 items). According to the cut-offs recommended from the World Health Organization’s Global Database on Body Mass Index, [44], BMI was computed from the height and weight measurements and categorized into four groups as: underweight (BMI < 18.5 kg/m^2^), normal (BMI 18.5–24.9 kg/m^2^), overweight (BMI 25–29.9 kg/m^2^) and obese (BMI ≥ 30 kg/m^2^). Smoking was identified by using a constructed variable of smoking status categorized into “never smoking”, “previous smoking” and “current smoking”. Data on marital status and age were provided from the National Population Registry. Marital status was categorized into two groups: “single/divorced/separated/widower” and “married/cohabitant/partnership”. Education level was specified in three categories: “primary school”, “high-school” and “university/college of four years or more”. Physical exercise was measured through two variables of light and hard duration as: “average of hours of both light and hard physical activity per week in the last year” and reflected “no” up to “moderate” levels of physical activity. Long-term illness (chronic disease) was evaluated by the question “do you suffer from any long-term illness or injury of a physical or psychological nature that impairs your functioning in everyday life?” where “long term” means at least one year. The response options were “yes/no”. The “no pain” group in the study population covered all subjects that did not satisfy the CWP criteria, including those that responded “yes” to the introductory question on pain but did not indicate the site of pain. The sex ratios were calculated by dividing female prevalence by male prevalence.

### Statistical analysis

Two models were used to examine predictors of CWP persistence. The examination of each was performed through logistic regression analyses resulting in odds ratios, with 95% confidence intervals. Model 1 captured the results from each variable of interest in a crude age- and sex-adjusted analysis. In model 2, all variables were included in one multivariable model. Because some variables were missing observations, only complete cases were included in the regression analyses. Missing observations concerned 1,157 respondents (23%) from the baseline sample. The analyses were examined for statistical interactions between sex and the independent variables. All data were analysed by using Stata 12.1 for Windows (Stata Corporation, USA).

### Ethics

This study was approved by the Board of Research Ethics in Health Region IV of Norway and the National Data Inspectorate.

## Results

Of the 28,367 subjects who completed the introductory question on chronic musculoskeletal pain, 28,313 subjects were included in HUNT2 and 27,574 were included in HUNT3. The overall mean age in the HUNT2 cohort was 50.1 years (SD 17.2), ranging from 19.1 to 101.1 years, while the mean age in the HUNT3 cohort was 53.1 years (SD 16.1), ranging from 19.0 to 100.8 years (data not shown). The mean age in the baseline sample (CWP in HUNT2) was 52.3 years (SD 11.4), varying between 19.5 to 85.9 years. The age distribution frequency of the baseline sample showed that the majority of the participants (59%) were aged 40 to 59 years. The mean baseline HADS depression and anxiety scores were 4.42 (SD 3.32) and 5.50 (SD 3.7), respectively, versus 3.49 (SD 3.1) and 4.24 (SD 3.4), respectively, in the study population (data not shown). Descriptive data on the variables of interest are described in Table 1.

**Table 1:** Sample characteristics of the study population and baseline sample (Widespread pain in HUNT2)

	**Study population**	*Baseline sample	**Female**	**Male**
	**N = 28,367 (%)§**	**N = 4,927 (%)§**	**N = 3,372 (68.4)**	**N = 1,555 (31.6)**
**Sex** Females (%)	16,260 (57)			
Males (%)	12,107 (43)			
**Age in years**, mean (SD)	47.6 (13.3)	52.3 (11.4)	51.7 (11.5)	53.7 (11.1)
20-39, (%)	8,397 (30)	733 (15)	558 (17)	175 (11)
40-59	24,664 (50)	2,892 (59)	1,996 (59)	896 (58)
60 years or older	5,707 (20)	1,302 (26)	818 (24)	484 (31)
**HADS score**; (%)				
HADS, (%) score < 8,	22,560 (81)	3,209 (67)	2,130 (65)	1,079 (71)
HADS-D, cut off score ≥ 8,	1,124 (4)	300 (6)	167 (5)	133 (9)
HADS-A, cut off score ≥ 8,	2,672 (10)	754 (16)	583 (18)	171 (11)
HADS-mixed	1,451 (5)	530 (11)	389 (12)	141 (9)
**Sleeping difficulties** (%)				
No problems	21,597 (81)	2,929 (63)	1,899 (59)	1,030 (70)
Problems	5,195 (19)	1,751 (37)	1,319 (41)	432 (30)
**Alcohol use** – abstainers (%)	2,546 (9)	552 (12)	444 (14)	108 (7)
0 times/month	7,256 (27)	1,516 (32)	1,156 (36)	360 (24)
1-7 times/month	15,790 (58)	2,398 (51)	1,488 (46)	910 (61)
8 ≥ times/month	1,718 (6)	240 (5)	123 (4)	117 (8)
**BMI** (%)				
Underweight	150 (1)	23 (1)	20 (1)	3 (0.2)
Normal	11,378 (40)	1,593 (32)	1,189 (35)	404 (26)
Overweight	12,606 (45)	2,262 (46)	1,394 (41)	868 (56)
Obesity	6,984 (25)	1,040 (21)	761 (23)	279 (18)
**Smoking status**, (%)				
Never smoked	12,912 (46)	1,759 (36)	1,315 (39)	444 (29)
Former smoker	8,249 (29)	1,612 (33)	940 (29)	672 (44)
Current smoker	6,984 (25)	1,517 (31)	1,089 (28.2)	428 (28)
**Exercise, moderate** (%)				
No exercise	16,123 (60)	2,974 (65)	2,044 (66)	932 (63)
Moderate exercise	10,731 (40)	1,575 (35)	1,035 (34)	540 (37)
**Education in years** (%)				
Primary school (%)	8,678 (31)	2,129 (45.6)	1,562 (48)	617 (41)
High school	12,649 (46)	1,946 (40.7)	1,229 (38)	717 (47)
College	6,435 (23)	653 (13.7)	472 (14)	181 (12)
**Marital status** (%)				
Not married/cohabitant	8,620 (30)	1,226 (25)	882 (26)	344 (22.2)
Married/cohabitant	19,694 (70)	3,690 (75)	2,482 (74)	1,208 (78)
**Chronic disease**, (%)				
No	21,018 (77)	2,156 (47)	1,527 (49)	629 (42)
Yes	6,116 (23)	2,418 (53)	1,566 (51)	852 (58)

§N in Study population will vary due to missing data.

*Calculated from the study population N = 28,367 (respondents of BOTH questionnaires Q1 and Q2 in HUNT2 and HUNT3).

N will vary slightly because of missing data.

The mean HADS scores in the HUNT2 study population were similar to the mean HADS scores in the HUNT2 cohort. Only 0.4% of the study population reported chronic musculoskeletal pain without specifying any pain location.

### Prevalence of persistent chronic widespread pain after 11 years

Of those reporting CWP in HUNT2 (i.e. baseline = 17%), 53% (n = 1,997) still reported CWP in the HUNT3 follow-up, and 74% of these were females. By comparison, the overall female proportion in the study population was 57%.

### Regression results

#### Crude analysis

From the health-related variables in the unadjusted analysis (see Table 2), symptoms of depression (OR: 1.28, 95% confidence interval (CI): 0.96–1.69) indicated a weak increase in CWP persistence. Symptoms of anxiety showed a 35% increased odds of CWP (OR: 1.35, 95% CI: 1.12–1.62), while symptoms of mixed anxiety and depression showed a 48% increased odds of CWP persistence (OR: 1.46, 95% CI: 1.19–1.82), versus the absence of anxiety and depression symptoms. Similarly, sleeping problems showed an increased CWP persistence compared with no sleeping problems (OR: 1.49, 95% CI: 1.30–1.71). Those with a chronic disease had almost twice the odds of CWP persistence compared to those without a chronic disease (OR: 1.89, 95% CI: 1.66–2.16).

**Table 2:** Odds ratios (ORs) with 95% confidence intervals (CIs) for persistent chronic widespread pain in HUNT3

**Baseline variables HUNT2**	**N = 3,770**	**CWP in HUNT3 N = 1,997**	**Model 1 – crude model**	**Model 2 – adjusted model**
	**N (%)**		**OR [95% CI]**	**OR [95% CI]**
Sex Female [ref.]	2,540 (67)	1,447 (74)	1.00	1.00
Male	1,230 (33)	520 (26)	0.53 [0.46-0.61]	0.51 [0.44-0.59]
Age, 20–39 yrs [ref.]	643 (17)	342 (17)	1.00	1.00
Age, 40–59 yrs (<40 yrs [ref.])	2,388 (63)	1,323 (66)	1.12 [0.94-1.34]	1.03 [0.85-1.25]
≥60 yrs	739 (20)	332 (17)	0.76 [0.62-0.94]	0.63 [0.50-0.81]
HADS (A/D <8 [ref.])	2,544 (67)	1,276 (64)	1.00	1.00
HADS Depression (D) ≥8	220 (6)	120 (6)	1.28 [0.96-1.69]	1.12 [0.84-1.50]
Anxiety (A) ≥8	578 (15)	343 (17)	1.35 [1.12-1.62]	1.24 [1.02-1.50]
Depression ≥8 AND Anxiety ≥8	428 (11)	260 (13)	1.48 [1.19-1.82]	1.18 [0.95-1.47]
Sleep problems, never [ref.]	2,544 (64)	1,195 (60)	1.00	1.00
Often	1,345 (36)	802 (40)	1.49 [1.30-1.71]	1.30 [1.12-1.51]
Smoke status, never smoking [ref.]	1,315 (35)	667 (33)	1.00	1.00
Ex. smoker	1,235 (33)	665 (33)	1.23 [1.05-1.45]	1.18 [1.00-1.39]
Current daily smoker	1,220 (32)	665 (33)	1.14 [0.97-1.33]	1.10 [0.93-1.31]
BMI normal 18.5-24.9 kg/m^2^ [ref.]	1,262 (33)	633 (32)	1.00	1.00
Underweight <18.5 kg/m^2^	21 (0.6)	7 (0.35)	0.46 [0.18-1.15]	0.43 [0.17-1.10]
Overweight 25–29.9 kg/m^2^	1,730 (46)	893 (45)	1.17 [1.01-1.36]	1.18 [1.01-1.37]
Obese ≥30 kg/m^2^	757 (20)	464 (23)	1.68 [1.39-2.02]	1.66 [1.37-2.01]
Alcohol-monthly use, abstainers [ref.]	375 (10)	205 (10)	1.00	1.00
0 time a month	1,139 (30)	615 (31)	0.94 [0.74-1.20]	0.97 [0.76-1.24]
1-7 times a month	2,046 (54)	1,077 (54)	0.95 [0.76-1.19]	1.01 [0.79-1.28]
8 times or more a month	210 (6)	100 (5)	0.85 [0.60-1.21]	0.90 [0.62-1.29]
Education, primary school, [ref.]	1,546 (41)	824 (41)	1.00	1.00
High school	1,661 (44)	900 (45)	1.03 [0.89-1.20]	1.10 [0.94-1.28]
University/college	563 (15)	273 (14)	0.76 [0.62-0.93]	0.86 [0.70-1.06]
Exercise, low/no [ref.]	2,487 (66)	1,338 (67)	1.00	1.00
Moderate	1,283 (34)	659 (33)	0.94 [0.82-1.07]	0.97 [0.84-1.12]
Marital status - single [ref.]	925 (25)	505 (25)	1.00	1.00
Married	2,845 (75)	1,492 (75)	0.92 [0.79-1.07]	0.94 [0.80-1.10]
Disease Chronic, no [ref.]	1,806 (48)	830 (42)	1.00	1.00
Yes	1,964 (52)	1,167 (58)	1.89 [1.66-2.16]	1.75 [1.53-2.01]

Model 1: Crude – age and sex adjusted.

Model 2: All variables included.

Only complete cases included.

Compared with never smokers, former smokers were at greater risk of CWP persistence (OR: 1.23, 95% CI: 1.05–1.45), while current smokers differed only marginally from never smokers (OR: 1.14, 95% CI: 0.97–1.33). Compared with study participants with normal weight, obese participants had higher odds of CWP persistence (OR: 1.68, 95% CI: 1.39–2.02). Drinking alcohol 8 times or more a month (OR: 0.85, 95% CI: 0.60–1.21) showed a non-substantial protective tendency when compared with abstainers. Participants 60 years or older had a lower odds of CWP persistence (OR: 0.76, 95% CI: 0.62–0.93) compared with the youngest age group (age 20–39). Also, those with an education at the university/college level had a lower odds of CWP persistence (OR: 0.76, 95% CI: 0.62–0.93) than those with a primary education only. Males had about half the odds of CWP persistence (OR: 0.53, 95% CI: 0.46–0.61) compared with females.

#### Adjusted analysis

In the adjusted analysis, symptoms of anxiety (OR: 1.24, 95% CI: 1.02–1.50) and mixed anxiety and depression (OR: 1.18, 95% CI: 0.95–1.47) only indicated a weak increase in CWP persistence. Sleeping problems remained a substantial predictor of CWP persistence (OR: 1.30, 95% CI: 1.12–1.51), compared with the lack of sleeping problems. Those with a chronic disease had a 75% increased odds of CWP persistence (OR: 1.75. 95% CI: 1.53–2.01) compared with participants without a chronic disease. Adjusted for the other possible predictors, overweight participants had a weak increased odds of CWP persistence (OR: 1.18, 95% CI: 1.01–1.32) while obese participants had a 66% increased odds of CWP persistence (OR: 1.66, 95% CI: 1.37–2.01) compared with normal weight individuals. Former smokers had a weak increase in CWP persistence (OR: 1.18, 95% CI: 1.00–1.39) compared with never smokers. Compared with the youngest age group (age 20–39), study participants 60 years or older showed a reduced odds of CWP persistence (OR: 0.63, 95% CI: 0.50–0.81), and males had about half the odds of CWP persistence (OR: 0.51, 95% CI: 0.44–0.59) compared to females. All results, both crude and adjusted models, are presented in Table 2.

### Comparison of HUNT3 non-participants and participants with CWP in HUNT2

Some participants experienced CWP in HUNT2, but did not attend HUNT3 (n = 2,451). To obtain descriptive measures, these HUNT3 non-participants were compared to the HUNT3 participants who had CWP in HUNT2, i.e. the baseline sample (n = 4,927). The mean age of non-participants was 54.4 years (SD 15.7). Compared to the baseline sample, non-participants were more likely to be obese, have sleeping problems and smoke daily. There were also group differences in age distribution, education, marital status and chronic disease. The results are presented in Table 3.

**Table 3:** **Comparison of measures of non-participants**^**a **^**in HUNT3 with CWP in HUNT2, and the baseline sample**^**b**^

	^**a**^**Non-participants HUNT3 N = 2,451(%)§**	^**b**^**Baseline sample HUNT2 N = 4,927 (%)§**
**Sex** Females (%)	1,634 (67)	3,372 (68)
Males (%)	817 (33)	1,555 (32)
**Age in years**, mean (SD)	54.4 (15.7)	52.3 (11.4)
20-39, (%)	513 (21)	733 (15)
40-59	964 (39)	2,892 (59)
60 years or older	974 (40)	1,302 (26)
**HADS score**; (%)		
HADS, (%) score < 8,	1,426 (63)	3,209 (67)
HADS-D, cut off score ≥ 8,	141 (6)	300 (6)
HADS-A, cut off score ≥ 8,	374 (16)	754 (16)
HADS-mixed	327 (14)	530 (11)
**Sleeping difficulties** (%)		
No problems	834 (53)	2,929 (63)
Problems	759 (47)	1,751 (37)
**Alcohol use** – abstainers (%)	396 (17)	552 (12)
0 times/month	775 (34)	1,516 (32)
1-7 times/month	1,017 (45)	2,398 (51)
8 ≥ times/month	91(4)	240 (5)
**BMI** (%)		
Underweight	19 (1)	23 (1)
Normal	279 (30)	1,593 (32)
Overweight	1,034 (43)	2,262 (46)
Obesity	634 (26)	1,040 (21)
**Smoking status,** (%)		
Never smoked	749 (31)	1,759 (36)
Former smoker	637 (27)	1,612 (33)
Current smoker	1,004 (42)	1,517 (31)
**Exercise, moderate** (%)		
No exercise	1,378 (66)	2,974 (65)
Moderate exercise	699 (34)	1,575 (35)
**Education in years** (%)		
Primary school (%)	1,251 (55)	2,129 (45.6)
High school	796 (35)	1,946 (40.7)
College	211 (9)	653 (13.7)
**Marital status** (%)		
Not married/cohabitant	941 (38)	1,226 (25)
Married/cohabitant,	1,509 (62)	3,690 (75)
**Chronic disease**, (%)		
No	873 (39)	2,156 (47)
Yes	1,345 (61)	2,418 (53)

§N will vary due to missing data.

^a^Participants with CWP attending HUNT2 cohort but not attending HUNT3.

^b^Baseline Sample (respondents with CWP in HUNT2 and responding to BOTH questionnaires.

Q1 and Q2 in HUNT2 and HUNT3.

### Statistical interaction analyses

Statistical interaction analyses indicated weak evidence of statistical interaction between sex and the other evaluated variables (p > 0.12).

## Discussion

In this large prospective cohort study, 53% of the participants with baseline CWP still had CWP at follow-up. According to the adjusted model, we found strong positive associations over time between persistent CWP and sleeping problems, being obese and having a chronic disease, while anxiety, mixed anxiety and depression symptoms, former smoking and being overweight showed weaker positive associations with persistent CWP 11 years later. Being a male and being 60 years of age were substantial protective factors against long-term persistent CWP.

### Strengths and weaknesses

#### Strengths

A major strength of this study was its large population base, its acceptable participation rate and its long-term follow-up. The study also contained a robust design and a lot of variables for comparing between different follow-up times. The pain measures were gauged with validated, well-known and standardized questionnaires that rely on self-reporting [1], which also would allow for reliable comparisons. By allowing the respondents to select individual body pain sites, we obtained a picture of the extent and the distribution of the pain by constructing and calculating the pain’s breadth. This study examined pain only by pain duration and pain location (i.e. CWP) but not by pain intensity or temporal features, and thus the study did not differentiate between intermittent, fluctuating or continuous pain, nor if the pain was disease related [45].

#### Weaknesses

Defining chronic pain only by duration and location, but not by pain intensity or severity may weaken the study’s optimality. In epidemiologic studies of pain, which are often based on self-report questionnaires rather than clinical findings, the complex nature of chronic pain represents a major challenge for the study’s validity. Another weakness is that the participation rate decreased in the last HUNT study, as it does in most longitudinal population-based cohort studies. The attrition was 15% and was most pronounced among males and younger adults. A non-participant study based upon the HUNT2 study revealed that being abroad and the lack of time for scoring were the main reasons for non-attendance for people aged 20–44 years [36]. Decreased participation rates might threaten the internal validity in population-based studies [46]. In this study, the non-participants were likely to be older and less educated, experience sleeping problem and a chronic disease, and smoke on a daily basis. In general, non-participation in HUNT3 was characterized by lifestyle factors regarding smoking and physical inactivity, along with higher prevalence of several chronic diseases [46], which is in line with our findings. However, the participation rate for age groups 60 – 80 years in HUNT 3 were 65% to 70% compared to the overall participation rate of 54%. We also believe that having the option to indicate bilateral pain at baseline, might have affected the sample size and thus the outcome. Meeting the ACR criteria of CWP by having chronic pain in all three major body areas paired with the option to indicate pain in both sides, may have resulted in a lower prevalence of baseline CWP and may have indicated a more severe pain condition. However, the baseline in this study covers all responders with CWP, including those with bilateral pain.

### Possible interpretations

Although this rather large study indicates that CWP occurs frequently in the general population, the conditions within CWP do not necessarily appear as invariable or unchanging, and with time, the individuals could move through various categories of chronic pain syndrome [47]. In this study a few factors point to pain persistence. Several factors not included in the HUNT2 study might explain why 53% of the baseline CWP persisted at follow-up. Lacking the possibility to indicate pain intensity and bilateral pain, which might indicate a level the pain condition’s severity, could be one factor. In addition, detailed information on social aspects might be of interest in this context. Likewise, more detailed information on medical treatment such as analgesics, which the HUNT2 survey roughly offered, or comorbid conditions, could be of interest for explaining pain persistence. Data on medication were, however, omitted due to incomplete responses.

Yet, a substantial number of subjects at baseline (47%) changed from having CWP at baseline to not having CWP at follow-up. The fact that a large proportion of the general population actually does resolve from CWP is additional knowledge for pain research and treatment. This finding is in contrast with other studies showing that the overall prevalence of CWP is stable over time and that pain is likely to persist if accompanied by, for example somatic symptoms and older age [31,32]. Our results may partly derive from investigating the general population in an 11-year longitudinal follow-up study. Another reason may be due to another important finding, namely that CWP prevalence decreased among those at 60 years and older. This means that participants in their 50s at baseline would accordingly be in their 60s at follow-up, thus entering an age group associated with decreasing CWP. According to the present study, aging was not associated with an increased likelihood of persistent CWP. Instead, aging appeared to be a protective factor against chronicity, despite the fact that some rheumatic diseases, for example, occur at older ages. Still, it make senses to assume that the high prevalence of CWP (17%) in this study may be related to the age distribution and the mean age of the HUNT cohort and the baseline sample, and the fact that CWP mainly affects middle-aged and older females and males [47,48]. These findings could indicate that CWP among the older population is poorly understood and mainly studied as a key symptom in fibromyalgia [39,47-50]. Associations between aging and pain are multidimensional, and at old(er) ages the problem of pain is considered to be highly complex due to multiple comorbidities [51]. Although multi-morbidity becomes more common with age, more than the half those with multi-morbidity and nearly two-thirds with physical–mental health comorbidity are younger than 65 [52]. Our study revealed that having other chronic diseases was the strongest predictor of CWP persistence. Sex differences in pain reporting have been ascribed to a range of factors: e.g. pain perception factors such as menstrual cycle variations in pain sensitivity in younger females [48], the greater likelihood for females to visit a physician and to report long-lasting pain, the lower pain threshold among females and their greater prevalence of having pain related to autoimmune disease [18]. Males and females at older ages also differ in many of these factors [48,53]. Likewise, age-related sex differences may be due to biological (e.g. gonadal hormones) and psychosocial factors (e.g. older people are more exposed to depression, life cycle changes, etc.), as well as multi-morbidity as a part of aging [51], which means a shift in attention from pain to other conditions. A previous study [47] reported that the prevalence of CWP peaks at midlife and then slowly decreases. Another study [32] found that the highest prevalence of CWP was among those of 59–74 years of age. In line with other studies, we found a female over-representation both in the baseline sample (2:1) and among the respondents with persistent CWP at follow-up (3:1). The most frequently stated ratio for chronic musculoskeletal pain and CWP in males and females is that females are about 1 ½ times more likely to experience both chronic musculoskeletal pain and CWP [49]. We found that only sleeping problems was associated with persistent pain in females, just as only former smoking was associated with persistent pain in males. Except from the sex ratio and in terms of prognostic factors, we did not find specific sex differences among those with CWP at baseline.

Depression and anxiety disorders are commonly reported co-morbid conditions in CWP [26,29,47,54-57]. These are usually based on cross-sectional and clinical samples rather than prospective community subjects. Clauw and Crofford [49] suggested that by examining CWP in population-based studies, the bias from using clinical patient samples would be removed and CWP would be much similar to other chronic pain syndromes. In our large population-based study, we hypothesized that symptoms of anxiety and/or depression would predict the persistence of CWP at the 11-year follow-up. However, we found only weak associations between symptoms of anxiety and mixed anxiety and depression and persistent CWP. Because depressive symptoms overlap with somatic symptoms in chronic pain conditions, depressive disorders could be over-diagnosed in people with chronic pain [58]. Likewise, it is possible that we were unable to reach those with severe pain or that the respondent’s pain could be perceived as mild despite the chronicity and breadth or underlying musculoskeletal disease. Although these conditions often cause severe pains, knowing the origin of the pain and/or having an opportunity to receive adequate treatment could influence how the subjects deal with suffering from a long-standing chronic pain condition. According to Dersh and colleagues [59], there are several reasons for considering chronic pain and mood disorders as distinct disorders even though the underlying patho-physiologies may share common factors. The onset of pain and mild psychological disturbances do not typically coincide, and each condition could be mutually independent for many individuals [59]. While those with chronic pain and psychological distress frequently tend to consult health services, not all those with chronic pain experience psychological distress as anxiety or depression [60], which might explain the weak associations in our study. Considering BMI, only obesity had a substantial effect on persistent CWP. According to several prospective studies, being overweight and obese are risk factors for chronic pain because weight gains increases bodily strains, especially strains in joints [61-63]. Our findings support a previous study reporting that CWP is significantly associated with obesity alone, but weakly associated with being overweight [63].

## Conclusion

The results demonstrated that many respondents (53%) in the general population maintained CWP, but a large proportion (47%) also resolved within an 11-year period. Persistent CWP was examined in association with important cofactors suggested by previous research. The study revealed strong positive associations over time with sleeping problems, obesity and chronic disease, while being 60 years or older was protective. Contrary to other studies, depression, anxiety and the frequency of alcohol consumption were not substantial predictors of outcome. Except from the sex differences in prevalence, the adjusted analyses revealed no prominent sex differences related to the predictors or protectors of CWP persistence. This large prospective population-based study brings new knowledge about what is important for long-term CWP persistence. Additional prospective population studies could reveal predictive factors for why many CWP individuals recover and why others do not recover from pain.

## Competing interests

The authors declare no conflict of interest.

## Authors’ contributions

IM was responsible for collecting and analysing data and manuscript drafting. RWG, OML and EAF contributed to the study’s conception, as well critical revision and manuscript drafting. JHB was involved in the data analysis and statistical interpretation, and contributed in particular with methodical and epidemiological support and critical revision and manuscript revising and drafting. All authors have made substantial intellectual contributions and read and approved the final manuscript.

## Pre-publication history

The pre-publication history for this paper can be accessed here:

http://www.biomedcentral.com/1471-2474/15/213/prepub
